# AlNAC4 Transcription Factor From Halophyte *Aeluropus lagopoides* Mitigates Oxidative Stress by Maintaining ROS Homeostasis in Transgenic Tobacco

**DOI:** 10.3389/fpls.2018.01522

**Published:** 2018-10-29

**Authors:** Jackson Khedia, Parinita Agarwal, Pradeep K. Agarwal

**Affiliations:** ^1^Academy of Scientific and Innovative Research, CSIR-Central Salt and Marine Chemicals Research Institute, Council of Scientific and Industrial Research, Bhavnagar, India; ^2^Division of Biotechnology and Phycology, CSIR-Central Salt and Marine Chemicals Research Institute, Council of Scientific and Industrial Research, Bhavnagar, India

**Keywords:** abiotic stress tolerance, *Aeluropus lagopoides*, AlNAC4, oxidative stress, transcription factors

## Abstract

NAC proteins are a large family of plant-specific transcription factors which regulate both ABA-dependent and -independent gene expression. These transcription factors participate in biotic and abiotic stress-response through intricate regulation at transcriptional, post-transcriptional and post-translational levels. In the present study, AlNAC4 transcription factor was isolated from a salt excreting halophyte *Aeluropus lagopoides*. The *AlNAC4* has an open reading frame of 936 bp, encoding a protein of 312 amino acid, with an estimated molecular mass of 34.9 kDa. The *AlNAC4* showed close homology to monocot NACs in the phylogenetic tree. *In silico* analysis revealed that AlNAC4 possess the characteristic A-E subdomains within the NAC domain. The AlNAC4 showed sixteen post-translational phosphorylation sites. The *AlNAC4* transcript was significantly upregulated with dehydration and H_2_O_2_ treatments, showing its role in osmotic and oxidative stress, respectively. The recombinant protein showed binding to mono as well as tandem repeats of NAC recognition sequence (NACRS) of the *erd1* promoter. This is the first report mentioning that overexpression of *AlNAC4* improved oxidative stress tolerance in tobacco transgenics. The transgenics maintained ROS homeostasis during H_2_O_2_ treatment. The transgenics showed regulation of stress-responsive genes including *CAT, SOD*, *LEA5*, *PLC3*, *ERD10B*, *THT1* and transcription factors like *AP2*, *ZFP* during oxidative stress.

**Key Message:** The AlNAC4 transcription factor from recretohalophyte Aeluropus showed regulation with abiotic stresses and binding to NACRS elements of *erd1* promoter. The *AlNAC4* tobacco transgenics showed improved growth with oxidative stress.

## Introduction

The growth and development of sessile autotrophic plants is negatively affected by adverse environmental conditions. Plants manage stress-response via morphological, physiological and biochemical changes, involving expression of functional and regulatory genes for sustainable biological function (Hirayama and Shinozaki, 2010). The transcription factors (TFs) play a pivotal role in complex signaling network by regulating a large number of downstream genes (Zhang et al., 2011). Approximately 5–7% of the total genes comprise TFs in plants, which have expanded largely in plant kingdom due to the complexity of plant metabolism (Shiu et al., 2005).

The NACs are plant-specific TFs, involved in various biological processes including abiotic and biotic stress responses via both ABA-dependent and -independent stress signal transduction pathways (Nuruzzaman et al., 2013). The NAC TF coined by the initials of NAM (*Petunia* no apical meristem; Souer et al., 1996), ATAF1/2 (*Arabidopsis thaliana* transcription activation factor) and CUC2 (*Arabidopsis* cup-shaped cotyledon) proteins (Aida et al., 1997). The NAC TF was originally identified from petunia (*Petunia hybrida*) as NAM (Souer et al., 1996), which plays a critical role in determining meristem and primordia positions. The N-terminal region, called NAC domain, is divided into five sub-domains A-E and is associated with various functions like nuclear localization, DNA binding and formation of homodimer or heterodimer with other NAC domain-containing proteins (Ooka et al., 2003; Olsen et al., 2005). The C-terminal region is highly variable, acting as transcription regulatory region, having either transmembrane motifs or protein binding activity (Seo et al., 2008). The potential of NAC TFs to perform wide range of functions is tightly regulated at transcriptional, post-transcriptional and post-translational levels. The NAC proteins show binding to NACRS (CATGTG) as multimers, for regulating the transcriptional activity of the NAC proteins (Tran et al., 2004). The NAC TFs are transcriptionally and post-transcriptionally regulated by ABREs (ABA-responsive elements) and DREBs (Dehydration-responsive element binding) and micro-RNAs or alternative splicing, respectively. The post-translational modifications like ubiquitination, dimerization, phosphorylation or proteolysis also regulate NAC TFs activity (Kaneda et al., 2009; Miao et al., 2016).

The Poaceae family includes cultivated staple food crops viz. rice, wheat, maize and a lot of efforts have been made toward understanding physiology, genetics and genomics of these crops for improving yield under varied climatic conditions, nutritional quality, disease manifestations/management etc. *Aeluropus lagopoides (L.) trin. Ex Thwis*, a C4 salt secreting perennial halophytic grass, belongs to family Poaceae and is a close wild relative of bread wheat. *A. lagopoides* grows commonly in salt marshes and survives at even 1 M NaCl (Gulzar et al., 2003). It is important to isolate and characterize stress-responsive TFs from halophytes; as halophytes with their distinct genetic constitution have developed potential to survive and complete their life cycle in high salt habitats. These genes can be genetically engineered in crops to upregulate a cascade of stress-responsive genes to enhance stress tolerance. In the present study, we have isolated an abiotic stress-responsive NAC TF and studied its role during multiple stress conditions.

## Materials and Methods

### Plant Material and Stress Treatments

*Aeluropus lagopoides* plants were collected from the [CSIR-CSMCRI salt farm, Bhavnagar, Gujarat (N21847′13.5″; E72807′25.7″), India] and the nodal cuttings of 2–3 leaf stage were grown in half-strength Hoagland hydroponic medium (Hoagland and Arnon, 1950) in plastic pots under 300–350 μmol m^-2^ s^-1^ of photosynthetically active light with 16/8 h light/dark cycle at 25°C in a growth chamber. For transcript analysis, plantlets (10–12 cm) were acclimatized for 7 days and following treatments were given: (i) 100 μM abscisic acid (ABA), (ii) dehydration by wrapping plants in dry tissue paper at room temperature, (iii) 250 mM NaCl and (iv) 20 mM hydrogen peroxide (H_2_O_2_). Another set of seedlings was maintained under control conditions in half-strength Hoagland medium. For all treatments, leaf tissue was collected from three biological replicates after 1, 3, 6, 12, 24, and 48 h of treatments and kept at -80°C until used for RNA isolation.

For stress tolerance study the seeds of tobacco WT and T_0_ tobacco transgenic lines transformed with *AlNAC4* gene (L33, L50 and L64) were germinated on Murashige and Skoog (1962) medium supplemented with NaCl (100, 200, and 300 mM) and mannitol (50, 100, 150, and 200 mM). The seeds of WT and T_0_ transgenics were also germinated on Whatman filter paper soaked in sterilized Milli-Q water containing 0, 10, and 20 mM H_2_O_2_. Different parameters like hypocotyl length, root length, relative water content were studied and, histochemical (DAB and NBT) and biochemical analysis (H_2_O_2_ content_,_ CAT and SOD activity) was carried out after 11 days of treatment.

The WT and hygromycin positive T_1_ transgenic seedlings were also transferred to Hoagland medium for 4 weeks. The uniform plants were treated with 0, 10, and 20 mM H_2_O_2_ for 3, 12, and 24 h to analyze the biochemical parameters (H_2_O_2_ content_,_ CAT and SOD activity). For all the treatments tissues from three biological replicates were collected for further analyses.

### Isolation and Cloning of *AlNAC4* cDNA From *A. lagopoides*

The NAC sequences from *Triticum aestivum* (AY625683.1), *Hordeum vulgare* (JX855805.1), *Oryza sativa* (DQ394702.1), *Sorghum bicolor* (KC253232.1) and *Zea mays* (JQ217429.1) were retrieved from NCBI and aligned by DNAMAN for designing degenerate primers (NACFA1, NAC R1, NACFA2 and NAC R2). The total RNA was isolated using Trizol-like reagent (Pyvovarenko and Lopato, 2011), treated with DNaseI (MBI Fermentas) followed by first-strand cDNA synthesis using RevertAid cDNA synthesis kit (Thermo Scientific). The PCR reaction was carried out using cDNA as template, 150 ng degenerate primers, 200 μM dNTPs and 2.5 U Taq DNA polymerase in 50 μl reaction at 94°C for 2 min, 1 cycle; 94°C for 1 min; 55°C for 1 min and 72°C for 2 min, 35 cycles and last 72°C for 7 min, 1 cycle. The amplicon was cloned in pJET 1.2/blunt plasmid vector (Thermo Scientific) and sequenced. The partial length gene sequence was confirmed by NCBI BLAST and 5′ and 3′ RACE (rapid amplification of cDNA ends) was carried out to get full-length sequence of AlNAC4. The NACJ5 F1 : PAoligo-dT, NACJ5 F2: PAR1, NACJ5 F3: PAR2 and NACJ5 R1, NACJ5 R2, NACJ5 R3 primers were used for 3′ RACE and 5′ RACE, respectively. The forward 5′ RACE primers were used as per Invitrogen (United States). The NAC amino acid sequences of different plants were used to construct a phylogenetic tree by DNASTAR Navigator software (version 11.0.0.64). The presence of conserved motifs was analyzed using MEME (Bailey et al., 2006). The sequences of the primers used in the present study are mentioned in **Supplementary Table S1**.

### Isolation of *AlNAC4* Genomic Clone

The *AlNAC4* genomic fragment was PCR amplified using gene-specific primers (AlNAC4EcoRI F and AlNAC4XhoI R primers) with *A. lagopoides* genomic DNA as template and 150 ng of each primer, 200 μM dNTPs, 2.5 U *Taq* DNA polymerase in a 50 μl reaction using following PCR conditions; 94°C, 5 min for 1 cycle; 94°C, 1 min; 55°C, 1 min and 72°C, 2 min for 35 cycles and last 72°C, 7 min for 1 cycle. The PCR product was gel eluted followed by ligation in the pJET1.2 vector and sequenced.

### Expression Analysis of *AlNAC4* Using Real-Time PCR

For expression analysis of *AlNAC4* and downstream genes of the tobacco transgenics, the RNA was isolated (as mentioned above). Five micrograms of RNA was treated with DNaseI and used for cDNA synthesis (Thermo Scientific). The cDNA (1:10 dilution) was used as a template for RT-PCR analysis. The *Aeluropus* and tobacco actin genes were used as internal control genes for *AlNAC4* expression and downstream gene expression, respectively (**Supplementary Tables S1**, **S2**). The RT-PCR of *AlNAC4* was performed using gene-specific primers (AlNAC4RT F and AlNAC4RT R, **Supplementary Table S1**), using the first strand cDNA from stress-treated leaf tissue and its corresponding control. To study the regulation of the downstream genes in transgenics, 15-days-old WT and hygromycin positive T_1_ transgenic seedlings were transferred to Hoagland medium for 4 weeks. The RNA was isolated from leaf tissue of WT and T_1_ transgenic plants treated with different H_2_O_2_ concentrations (0, 10, and 20 mM) for 24 h. List of the primers used for downstream genes study is given in **Supplementary Table S2**.

The PCR reaction was carried out using SYBR green jumpstart Taq ready mix (Sigma–Aldrich) and 75 ng of each gene-specific primers in CFX96 PCR system (BioRad, United States) at following conditions: 94°C, 2 min for 1 cycle; 94°C, 30 s, 55°C, 30 s and 72°C, 30 s for 45 cycles; 72°C, 7 min for 1 cycle. The specificity of PCR amplification was checked at the end of the PCR cycles, by melt curve analysis. Each reaction was replicated three times and relative-fold expression was calculated by the comparative C*_t_* (2^-ΔΔ*Ct*^) method using actin as an internal reference gene (Livak and Schmittgen, 2001). The expression values are mentioned as the mean ± standard deviation.

### Transactivation Assay of AlNAC4

A yeast one-hybrid assay was performed to study the transcriptional activation of the AlNAC4 protein (Shukla et al., 2015). *AlNAC4* cDNA was amplified using primers (AlNAC4EcoRI F and AlNAC4SalI R, **Supplementary Table S1**) with the *Eco*RI and *Sal*I flanking restriction sites, respectively, and cloned in a pGBKT7 vector (Clontech). *AlNAC4* and vector alone plasmids were transformed separately into yeast strain AH109 (Clontech) and grown on synthetic dropout medium lacking tryptophan (SD/-Trp). HIS3 activity was assessed by a viability test on a histidine lacking medium (SD/-Trp/-His/-Ura). The LacZ activity was analyzed by the galactosidase filter lift assay (Ma et al., 2009).

### Cloning of *AlNAC4* in *E. coli* Expression Vector and Purification of the Recombinant Protein

The *AlNAC4* ORF was PCR amplified using AlNAC4EcoRI F and AlNAC4XhoI R primers flanked with *Eco*RI and *Xho*I restriction sites, respectively, and cloned in the pET-28a expression vector (Novagen). The recombinant (pET28a-*AlNAC4*) plasmid and vector alone were transformed in the BL21 (DE3) star *Escherichia coli* strain. The recombinant plasmid transformed in BL21 (DE3) star *E*. *coli* strain was induced with 1 mM IPTG and cells were harvested after 2, 4, and 6 h of induction. Finally, the recombinant protein was purified to homogeneity using the 2 h harvested cells under the native condition using the Ni-NTA Fast Start Kit (Qiagen) following the manufacturer’s protocol. The AlNAC4 protein was detected by anti-6x-His antibody (Qiagen), followed by alkaline phosphatase conjugated secondary antibody; the signals were developed AP conjugate substrate kit (Bio-Rad).

### DNA Probes and Gel Mobility Shift Assay

For electrophoretic mobility shift assay (EMSA), two sets of complementary oligonucleotides for NACRS in tandem (51 bp, NACBST) and NACRS having flanking sequence (69 bp, NACBS) were designed from the *Arabidopsis thaliana* EARLY RESPONSIVE TO DEHYDRATION STRESS 1 (*erd1*) promoter (Tran et al., 2004). Two micrograms of complementary oligonucleotides were annealed using the annealing buffer (100 mM Tris-HCl, 5 mM NaCl and 10 mM EDTA) by incubating at 60°C for 5 min and 37°C for 15 min. The binding reaction was carried out in 25 μl reaction volume using 80 ng of probe, 300–2400 ng of purified protein and 4 μg glycogen in binding buffer (15 mM HEPES, 35 mM KCl, 1 mM EDTA pH 8.0, 1 mM DTT, 1 mM MgCl_2_ and 6% glycerol) at room temperature for 20 min (Gupta et al., 2010). The reaction was fractionated on non-denaturing acrylamide gel (10% acrylamide, 0.5x TBE, 5% glycerol) and the gel was stained with ethidium bromide. The MCS of pBSK+ vector was used as negative control (pBSK+ MCS F and pBSK+ MCS R; primers **Supplementary Table S1**). The NACBS was also mutated by replacing CATGTG nucleotide with AAAAAA and similarly AACA nucleotide with TTAA in NACBS F, respectively.

### Plant Transformation Vector Construction and Tobacco Transformation

The *AlNAC4* cDNA was PCR amplified by AlNAC4 F EcoRI and AlNAC4 R KpnI primers and cloned in *Eco*RI/*Kpn*I sites of pRT101 vector. The pCAMBIA1301 binary vector *Hind*III site was used for cloning expression cassette containing 35S:*AlNAC4*:PolyA and mobilized into *Agrobacterium tumefaciens* (LBA4404). The *Agrobacterium* cells comprising the 35S:*AlNAC4*:PolyA was used to transform *Nicotiana tabacum* L. cv. Petit Havana leaf disks according to Horsch et al. (1985).

### Confirmation of Transgenic Plants

The β-glucuronidase reporter gene staining kit (Sigma, United States) was used to visualize glucuronidase gene (GUS) activity in leaf tissue. Genomic DNA was isolated from different T_0_ lines using CTAB buffer (Doyle and Doyle, 1987) and PCR was carried out to confirm the integration of transgene using the glucuronidase (gus A F and gus A R), hygromycin (hptII F and hptII R) and gene-specific (AlNAC4RT F and AlNAC4RT R) primers (**Supplementary Table S1**).

Semi-quantitative RT-PCR was used for the determination of the transcript level of *AlNAC4* gene in the transgenic plant. The total RNA was isolated from WT and transgenic lines. The cDNA template (1:10 dilution) with gene-specific (AlNAC4RT F and AlNAC4RT R, **Supplementary Table S1**) and tobacco actin primers (internal control, NtActin F and NtActin R, **Supplementary Table S2**) were used for semi-quantitative RT-PCR analysis. An agarose gel electrophoresis analysis of the PCR products showed the *AlNAC4* mRNA expression in transgenic lines.

The copy number of the transgene was determined by Real-time PCR. The Real-time PCR was optimized for GUS and NRA genes (Nitrate reductase, NCBI accession no. X06134) primers. The PCR reaction was carried out using 7.5 ng primers in 20 μl reactions using SYBR green jumpstart Taq ready mix (Sigma–Aldrich) on 1, 10, and 100 ng of genomic DNA from T_1_ transgenic lines and the standard curve was plotted using threshold cycle (C_t_) value to determine reaction efficiencies for calculating copy number ratio of GUS to NRA (Shepherd et al., 2009) using following formula:

$$Ratio(GUS:NRA)={\left\{1+{\left(E\right)}^{C_{t}}\right\}}_{GUS}/{\left\{1+{\left(E\right)}^{C_{t}}\right\}}_{NRA}$$

### Analysis of Physiological and Biochemical Parameters of Transgenic in Response to Hydrogen Peroxide Treatment

#### *In vivo* Localization of O_2_^•-^ and H_2_O_2_

The *in vivo* localization and quantification of O_2_^•-^ and H_2_O_2_, were performed according to Shukla et al. (2012).

#### Quantification of H_2_O_2_ Content

The H_2_O_2_ content in leaf samples was measured as described by Sanadhya et al. (2015a). Leaf tissue extract was prepared with cold acetone to determine the H_2_O_2_ levels. 1 ml of the extract was mixed with 0.5 ml of 0.1% titanium dioxide in 20% (v:v) H_2_SO_4_ and the mixture was centrifuged at 6,000 *g* for 15 min. The intensity of the yellow color of the supernatant was measured at 415 nm and the concentration of H_2_O_2_ was calculated against the standard curve.

#### Determination of Enzyme Activities

Leaf tissue (1–2 g) from WT and transgenic plants were homogenized in 50 mM phosphate buffer, pH 7.0, containing 1% polyvinylpyrrolidone. The homogenate was centrifuged at 10,000 *g* for 10 min and the supernatant obtained was used as enzyme extract.

##### Superoxide dismutase (EC1.15.1.1)

Superoxide dismutase activity was measured as reported in Dhindsa et al. (1981) with minor modification, based on its potential to inhibit the photoreduction of NBT. The 3 ml reaction mixture contained 100 mM phosphate buffer, pH 7.5, 200 mM methionine, 2.25 mM NBT, 60 μM riboflavin, 3.0 mM EDTA, and 50 μl enzyme extract. The reaction was carried out at room temperature for 15 min under bright light (2 × 15 W fluorescent lamps), and absorbance was recorded at 560 nm. The log A_560_ was plotted as a function of the volume of enzyme extract (Giannopolitis and Ries, 1977), and the volume of enzyme extract corresponding to 50% inhibition of the reaction was considered as one enzyme unit (Beauchamp and Fridovich, 1971).

##### Catalase (EC1.11.1.6)

Catalase assay was carried out by measuring the initial rate of disappearance of H_2_O_2_ as reported by Dhindsa et al. (1981). The 3 ml reaction mixture contained 100 mM phosphate buffer, pH 7.5, 75 mM H_2_O_2_, and 50 μl enzyme extract. The decrease in H_2_O_2_ concentration was observed as a decline in O.D. at 240 nm using Epoch spectrophotometer (Biotek, India). The activity was expressed in units (1 unit defines the conversion of one mole of H_2_O_2_/minute).

### Statistical Analysis

Each experiment was repeated thrice, the mean values and standard deviations were calculated using Microsoft Excel. Analysis of variance was calculated using Fishers Least Significant Difference (LSD) by Infostat software at *P* ≤ 0.05 to determine the significance of difference between the means of control and different stress treatments. Mean values of treatments that were significantly different from each other were indicated by different alphabets.

## Results

### Identification and Sequence Analysis of *AlNAC4* Gene

An amplicon of 300 bp, having a conserved region of NAC TF, was amplified using degenerate primers. Later, the gene was made full-length by RACE. The 5′ and 3′ RACE, using gene-specific primers, resulted in the amplicons of 352 and 779 bp, respectively. The *AlNAC4* cDNA (NCBI accession number KY569078) had an ORF of 936 bp and 191 bp and 202 bp 5′ and 3′ untranslated region, respectively. The *AlNAC4* encodes a protein of 312 amino acid (aa), with the calculated molecular weight of 34.9 kDa and isoelectric point (pI) of 6.11.

**FIGURE 1:**
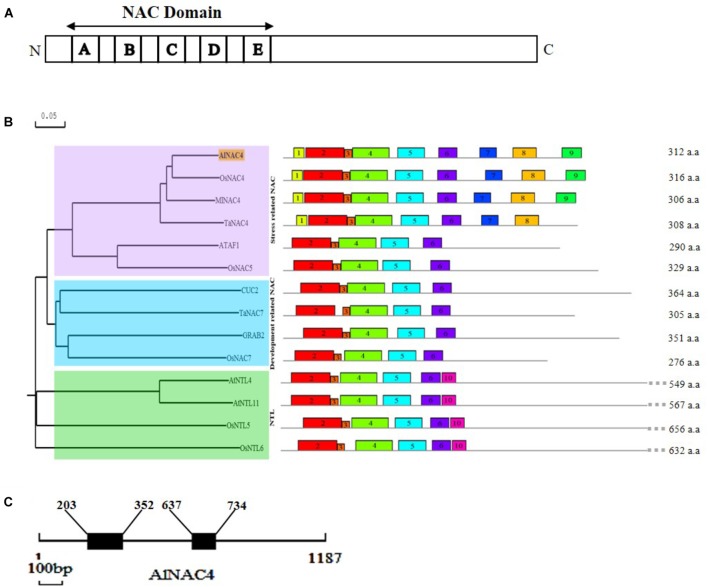
*In silico* analysis of AlNAC4 sequence. **(A)** Schematic representation of AlNAC4 protein with A-E sub-domains, **(B)** the relationship among NAC proteins is represented using N-J phylogenetic tree constructed by DNAMAN software. Scale represents the branch length. The sequences used from OsNAC4 (AB028183.1), MlNAC4 (KM017003.1), TaNAC4 (GQ985329), ATAF1 (CAA52771.1), OsNAC5 (AB028184.1), CUC2 (BK007973.1), TaNAC7 (HM027572), GRAB2 (AJ010830), OsNAC7 (BAA89801.1), NTL4 (NM_111885), NTL11 (NM_120523), OsNTL5 (NM_001069053), and OsNTL6 (NM_001055094). Conserved motifs were identified by MEME software (multilevel consensus sequences for the MEME defined motifs are listed in **Table 1**), **(C)** diagrammatic representation of the position of introns (203–352; 637–734bp) and exons of *AlNAC4*. The scale underneath the diagram represents 100 bp.

The AlNAC4 protein shows the presence of highly conserved 153 aa long NAC domain (21–174 aa) comprising of A (20 aa), B (17 aa), C (35 aa), D (28 aa), E (15 aa) sub-domains at N-terminal and a highly variable C-terminal region (**Figure 1A** and **Supplementary Figure S1**). The MEME predicted conserved motifs are presented in **Table 1**. The PROSITE analysis revealed seven protein kinase C phoshphorylation sites (10–12, 81–83, 116–118, 130–132, 160–162, 223–225, 245–247 aa), seven casein kinase II phosphorylation sites (30–33, 81–84, 105–108, 207–210, 217–220, 228–231, 245–248 aa), two amidation sites (10–13 and 155–158 aa), two N-glycosylation motifs (89–92 and 221–224 aa), four myristoylation sites (90–95, 115–120, 121–126, 287–292 aa) and single cAMP- and cGMP-dependent protein kinase phosphorylation sites (157–160 aa). The AlNAC4 amino acid sequence showed 81, 78, and 75% identity to MlNAC4, OsNAC4 and TaNAC4, respectively, indicating relatively high homology with only monocot NAC members, whereas no close homology was found with the dicot NAC members. Phylogenetic relationships among some NAC proteins show that AlNAC4 gets clustered with stress-responsive MlNAC4, OsNAC4 and TaNAC4 proteins of the stress-related NAC (SNAC) group III (**Figure 1B**).

**Table 1:** The multilevel consensus sequences of the MEME predicted motifs in different NAC genes.

Motif No.	Sequence	Amino acids	*E*-value
1	RRDAEAELNLP	11	5.4 e^-009^
2	GFRFHPTDEELVVYYLCRKVAGQPLPVPIIAEVDLYKLEPW	41	4.0 e^-303^
3	DLPEKALF	8	1.7 e^-010^
4	GEKEWYFFSPRDRKYPNGSRTNRATGTGYWKATGKDKPI	39	6.2 e^-298^
5	VGMKKTLVFYSGRAPRGVKTNWVMHEYRL	29	3.7 e^-274^
6	LDEWVLCRIFNKKGNGEKVG	20	2.5 e^-095^
7	DTMSDSFQTHDSDIDNAS	18	7.9 e^-015^
8	NGMVTVKEDNDWFTGLNFDELQASY	25	1.3 e^-025^
9	GYLQSISSPQMKMWQTILPPF	21	4.1 e^-019^
10	QYGAPYVEEEWEEED	15	5.6 e^-014^


*The underlined amino acids are not conserved. The motif logo is provided in the **Supplementary Figure S2**.*

For the analysis of *AlNAC4* genomic clone, 1,187 bp fragment (NCBI accession number KY569079) was PCR amplified using gene-specific primers from *A. lagopoides* genomic DNA. The genomic DNA sequence was compared with the cDNA sequence to identify the exons and introns positions. The *AlNAC4* genomic clone consists of three exons (1–202, 353–636, and 735–1,187 bp) and two introns (203–352 and 637–734 bp, **Figure 1C**).

### The Expression of *AlNAC4* Gene at the Transcript Level

**FIGURE 2:**
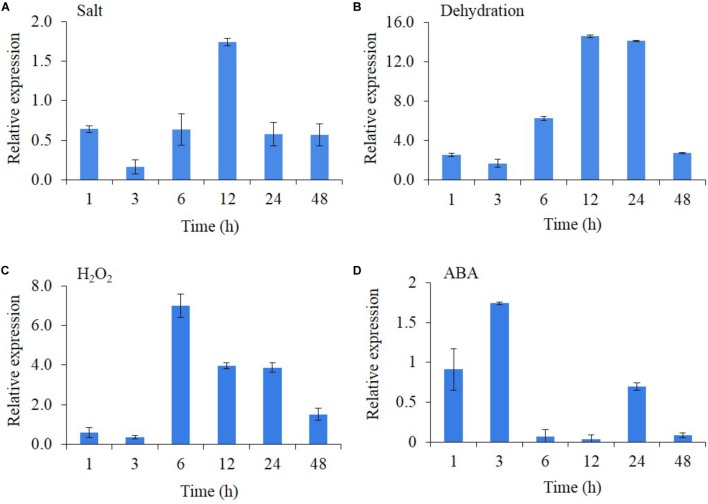
Transcript analysis of *AlNAC4* gene in response to 250 mM NaCl **(A)**, dehydration **(B)**, 20 mM H_2_O_2_ **(C)** and 100 μM ABA **(D)**. The experiments were replicated three times and the mean values and standard deviations are represented in bars.

The transcript expression of *AlNAC4* was studied in the presence of different stresses and stress-related phytohormones (**Figures 2A–D**). The results showed that *AlNAC4* expression was regulated by salinity, dehydration, H_2_O_2_ and ABA treatments. The salinity resulted in 1.75-fold transcript accumulation at 12 h duration only. The transcript showed strong expression with dehydration treatment reaching 2.6-fold expression as early as 1 h and showing a maximum transcript expression of 14.1 and 14.6 at 12 and 24 h duration. However, the transcript expression decreased at 48 h (2.7-fold). The H_2_O_2_ treatment also showed higher transcript expression, wherein the maximum expression was observed (7-fold) at 6 h and further reduced to 1.5-fold at 48 h. The ABA (100 μM) showed a maximum induction of only 1.74-fold at an early time point of 3 h.

### Transcriptional Activity and DNA-Binding Assay of AlNAC4 Protein

**FIGURE 3:**
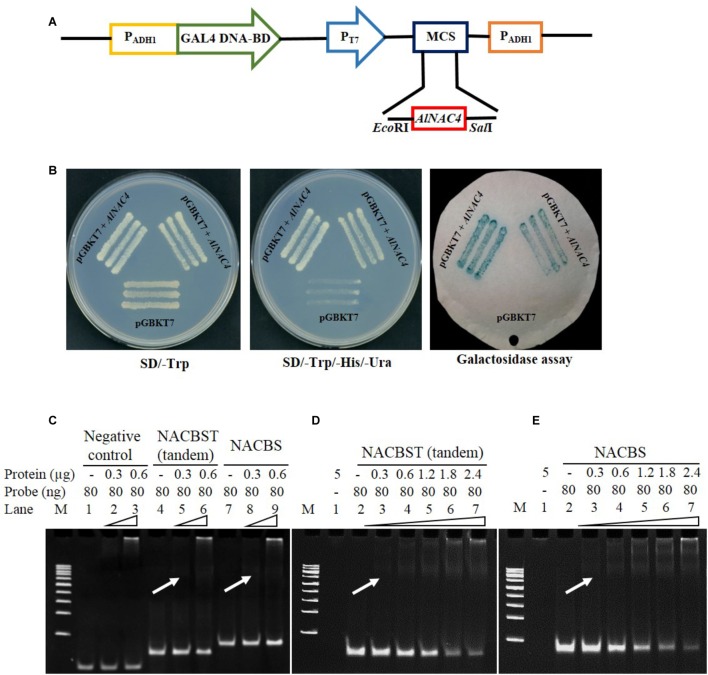
**(A)** Schematic representation of the *AlNAC4* ORF cloned in pGBKT7 vector, **(B)** transactivation assay for *AlNAC4* gene, (i) Transformed yeast cell (AH109) containing pGBKT7 + *AlNAC4* and pGBKT7 alone grown on SD/-Trp medium, (ii) Transformed yeast cell (AH109) containing pGBKT7 + *AlNAC4* showing growth on SD/-Trp/-His/-Ura medium, while only pGBKT7 did not grow, (iii) Yeast cells transferred on filter paper showed β-galactosidase activity using X-gal staining. Electro Mobility Shift Assay of AlNAC4 protein with heterologous, NACBST (tandem) and NACBS probes showing specific binding with NACBST and NACBS **(C)**. The detailed kinetics of AlNAC4 protein with NACBST (tandem, **D**) and NACBS **(E)** probes.

The transcriptional activity of the AlNAC4 protein was studied using a yeast GAL4 system. The GAL4 DNA-binding domain-*AlNAC4* recombinant plasmid was transformed into AH109 yeast strain. The yeast cells activated the transcription of the reporter gene His3 and LacZ, as was evident by the growth of yeast cells on SD/-His medium, and further, development of blue color in X-gal solution (**Figures 3A,B**), confirmed its activity as a TF.

The AlNAC4 recombinant protein of 34.9 kDa was induced with 1 mM IPTG for different time periods. The protein showed maximum induction at 2 h; therefore, the protein was induced for 2 h and purified to near homogeneity (**Supplementary Figures S3A,B**). The purified recombinant protein (without thrombin cleavage) was confirmed by Western analysis using 6x-His antibody (**Supplementary Figure S3C**). The purified protein was used to study the binding of the AlNAC4 protein with NACBST having three tandem repeats and NACBS having single repeat by EMSA. The heterologous probe (MCS of pBSK+) did not bind to the AlNAC4 recombinant protein (**Figure 3C**). The recombinant protein showed binding to both the oligos of the *erd*1 promoter. The strength of binding increased with increasing amount of recombinant protein from 300 to 2400 ng and 80 ng probe (**Figures 3D,E**). The mutated sequence did not show binding with AlNAC4 protein (data not shown).

### Analysis of Transgenic Lines

#### Confirmation and Transgene Integration in T_0_ Tobacco Transgenics

**FIGURE 4:**
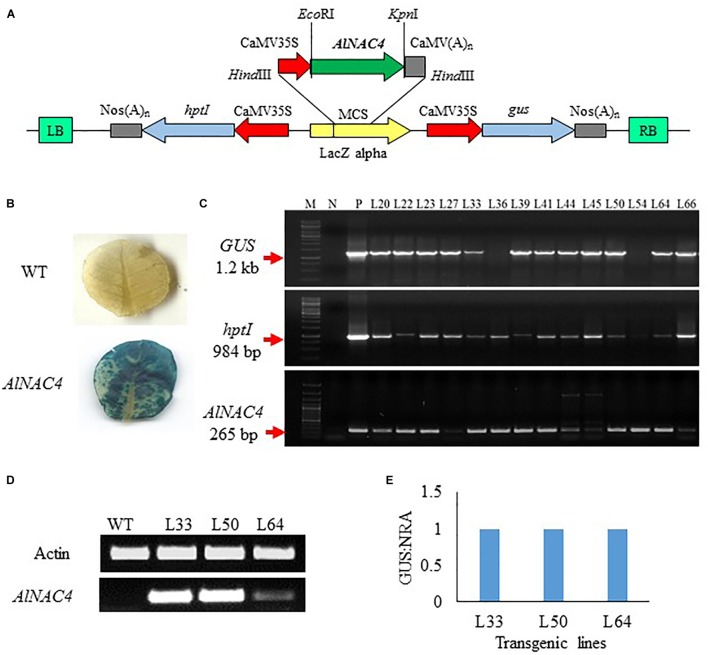
**(A)** Schematic representation of the pCAMBIA1301-35S: *AlNAC4* construct used for *Nicotiana tabacum* transformation, **(B)** GUS histochemical assay of wild type (WT) and *AlNAC4* transgenic leaves, **(C)** PCR confirmation of transgenic lines by *gus* (Glucuronidase, 1200 bp), *hptI* (hygromycin resistant, 984 bp) and *AlNAC4* (265 bp) gene specific primers, M denotes 1 kb ladder for upper two panel and 100 bp plus ladder for lower panel, N denotes wild type (WT) tobacco, **(D)** transcript level of *AlNAC4* gene in transgenic lines (L33, L50 and L64) and wild type (WT) plants via semi quantitative RT-PCR, **(E)** *AlNAC4* gene copy number analysis by Real-Time PCR.

To validate the function of *AlNAC4* gene in stress tolerance, the cDNA was cloned under the CaMV 35S promoter in pCAMBIA1301 (**Figure 4A**) and transformed into tobacco via *Agrobacterium tumefaciens*-mediated transformation. Sixty seven putative transgenic lines of *N*. *tabacum* were selected on hygromycin-containing medium and subsequently 20 of them showed positive GUS assay (**Figure 4B**). Some plants showed proper blue color in leaves while in others the scattered blue spots were seen, whereas no visual staining was observed in WT leaves. The GUS positive transgenic lines were confirmed by PCR using the presence of *GUS*, *hptII* and *AlNAC4* genes. Ten transgenic lines showed the single product of the expected size of all the genes (**Figure 4C**).

Three transgenic lines (L33, L50, and L64) and WT plants were checked by reverse transcriptase PCR to confirm the expression of transgene mRNA. Transgenic lines showed expression of the *AlNAC4* gene, but in the WT plants the corresponding band was not observed (**Figure 4D**). The presence of transcripts indicated that transcription initiation and termination of *AlNAC4* mRNA occurred as expected.

Transgene integration analysis was carried out using RT-PCR. The quantitative analysis revealed that L33, L50, and L64 had a GUS: NRA ratio 1.0, thus confirming the single-copy insertion (**Figure 4E**).

The T_0_ transgenic plants showed no morphological or growth differences at vegetative and floral stages and was similar to WT plants. Seed set in both transgenics and WT plants was also similar (**Supplementary Figures S4A,B**).

#### Analysis of T_1_ Transgenics for Stress Treatments

##### Salinity and dehydration stress

**FIGURE 5:**
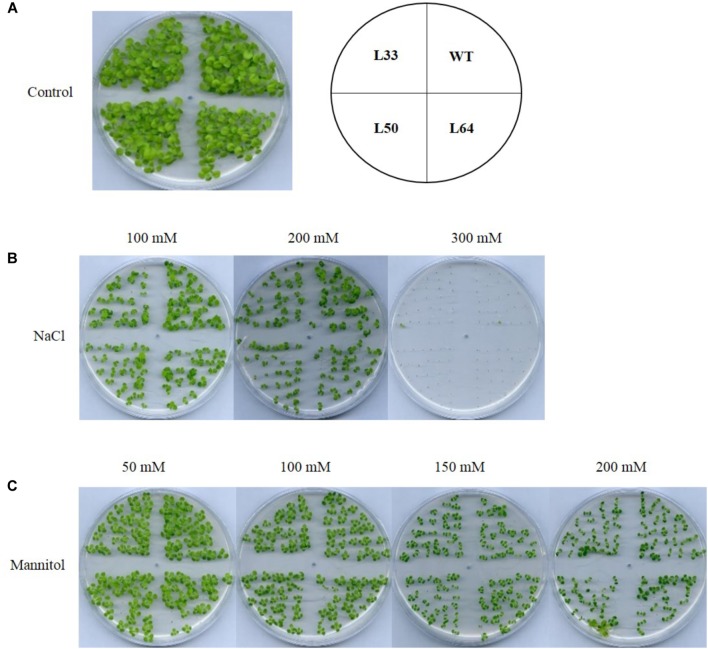
Seed germination analysis of wild type (WT) and 35S: *AlNAC4* transgenic (L33, L50, and L64) tobacco at 30 days at various stress supplemented medium, **(A)** control, **(B)** NaCl, and **(C)** mannitol.

The T_1_ transgenic progeny was studied to establish the stress tolerance potential of tobacco transgenics overexpressing *AlNAC4* gene. The WT and transgenics seeds (T_0_) showed similar germination rate and seedling growth in control conditions as well as in salinity and dehydration conditions (**Figures 5A–C**).

##### Oxidative stress

**FIGURE 6:**
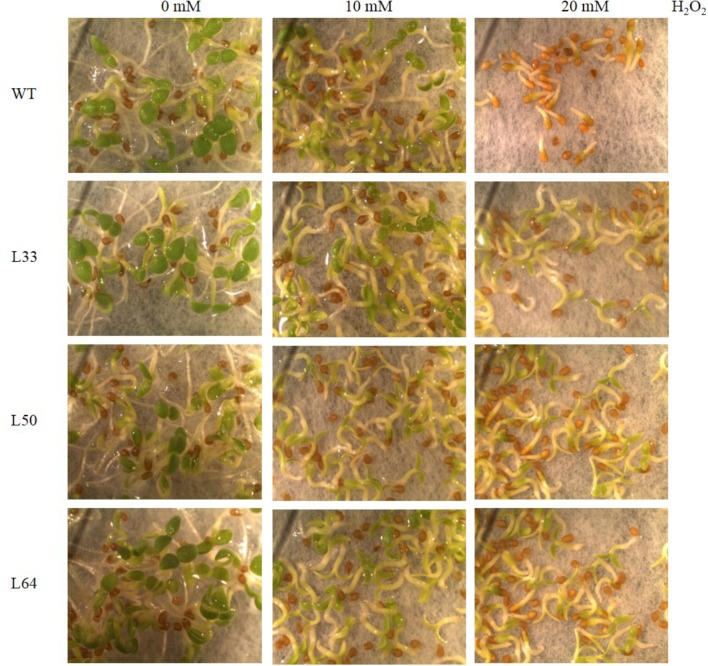
Stereomicroscopic study of seed germination of wild type (WT) and 35S: *AlNAC4* transgenic (L33, L50, and L64) tobacco on 0, 10, and 20 mM H_2_O_2_ at 6th day. The transgenic seeds showed significantly better growth at 20 mM H_2_O_2_.

The WT and transgenic lines showed similar germination rate and growth at 0 and 10 mM H_2_O_2_ after 6 days. While WT and T_1_ transgenic lines showed differential germination rate and growth after 6 days at 20 mM H_2_O_2_ (**Figure 6**).

**FIGURE 7:**
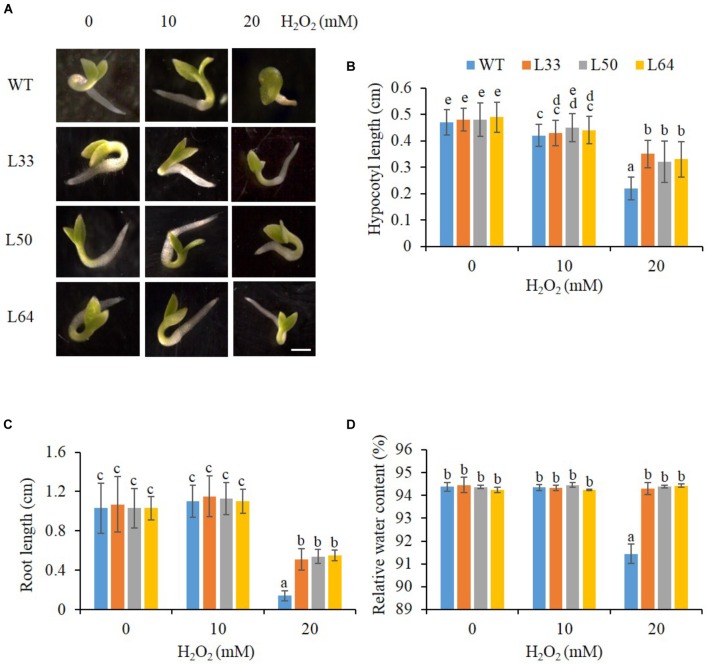
**(A)** Stereomicroscopic study of seed germination of wild type (WT) and 35S: *AlNAC4* transgenic (L33, L50 and L64) tobacco plants on 0, 10 and 20 mM H_2_O_2_ at 11th day. Scale bar 0.5 cm. Analysis of growth parameters **(B)** hypocotyl length, **(C)** root length and **(D)** relative water content (RWC) of T_1_ transgenic seedlings germinated in H_2_O_2_ stress (0, 10, and 20 mM). The experiment was repeated three times, and the mean values significantly different at *P* ≤ 0.05 within treatment are indicated by different alphabets.

The transgenic lines (L33, L50, and L64) showed enhanced growth (**Figure 7A**), with longer hypocotyl and roots as compared to WT seedlings with 20 mM H_2_O_2_ stress (**Figures 7B,C**). There was a slight difference in hypocotyl length in WT and transgenics at 10 mM H_2_O_2_ compared to the control condition, whereas no difference was observed in root length. An enhanced RWC percentage was exhibited in all transgenic seedlings at 20 mM H_2_O_2_ as compared to WT seedlings (**Figure 7D**).

#### Physio-Biochemical Analysis of Transgenics on H_2_O_2_ Treatment

##### Seedling stage

**FIGURE 8:**
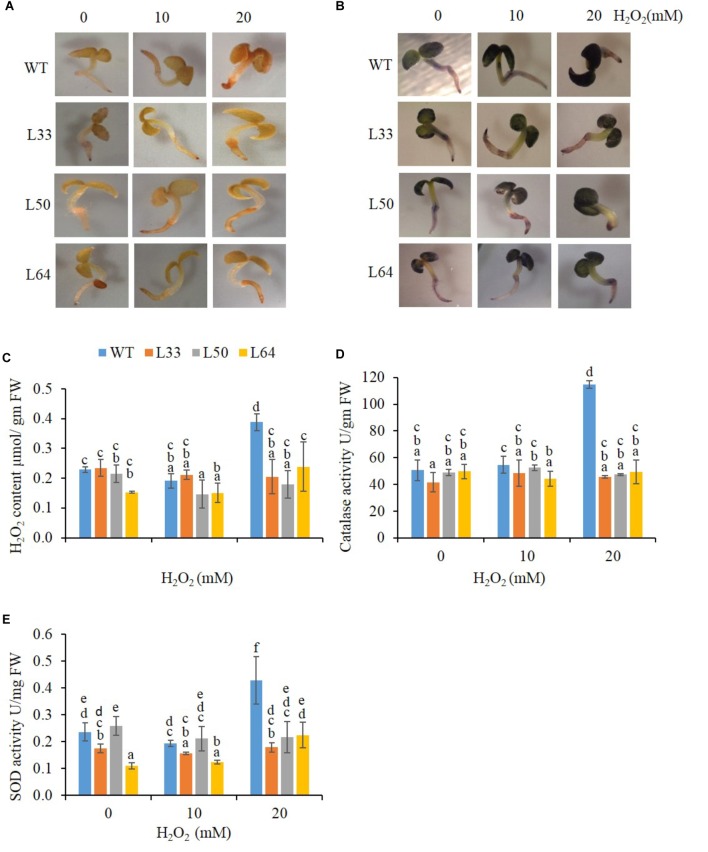
Biochemical study of wild type (WT) and transgenic (L33, L50, and L64) seedlings of tobacco at 11th day. *In vivo* localization of H_2_O_2_ **(A)** and O_2_**^-^ (B)**, quantification of H_2_O_2_ **(C)** and activity of CAT **(D)** and SOD **(E)** in the presence of 0, 10, and 20 mM H_2_O_2_ treatments. Values are represented as mean ± SD (*n* = 3) and marked with different alphabets to indicate significant difference at *P* ≤ 0.05 probability.

The WT and transgenic seedlings (11-day-old) showed similar staining at 0 and 10 mM H_2_O_2_ in the presence of DAB and NBT solutions. However, at 20 mM H_2_O_2_, WT seedlings accumulated more brown (indicator of H_2_O_2_) and blue-colored spots (indicator of O_2_^-^) in comparison to transgenics (**Figures 8A,B**). The WT seedling showed significantly higher accumulation of H_2_O_2_ (0.39 μmol/gm FW) at 20 mM H_2_O_2_ treatment (**Figure 8C**). The WT seedlings also showed higher anti-oxidative enzyme (CAT and SOD) activity (114.78 U/gm FW and 0.43 U/mg FW, respectively) at higher concentration of H_2_O_2_. While in transgenic seedlings (L33, L50, and L64), significantly lower H_2_O_2_ (0.21, 0.18, and 0.24 μmol/gm FW, respectively) as well as CAT (45.69, 47.35, and 49.29 U/gm FW, respectively) and SOD (0.18, 0.22, and 0.23 U/mg FW, respectively) activity was observed at 20 mM H_2_O_2_ stress (**Figures 8C–E**). The transgenic seedlings showed no significant difference in anti-oxidative levels at various H_2_O_2_ concentrations (0, 10, and 20 mM).

##### 1-month-old plant stage

**FIGURE 9:**
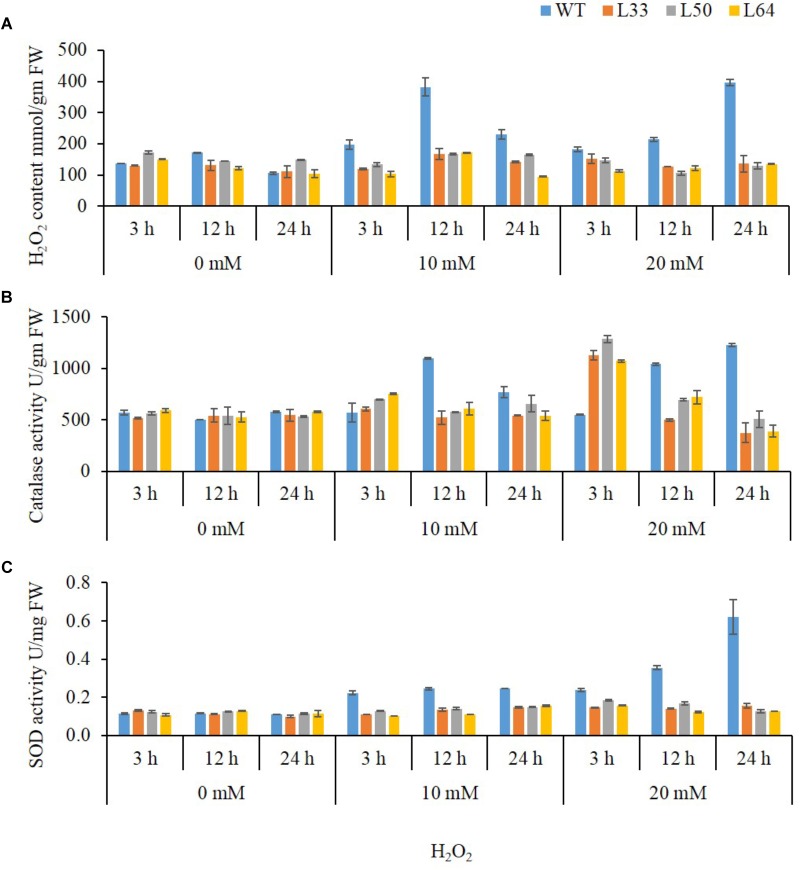
Biochemical study of 4-week-old wild type (WT) and transgenic tobacco (L33, L50, and L64) plants at different concentrations of H_2_O_2_ for 3, 12, 24 h. Quantification of H_2_O_2_ **(A)** content and activity of CAT **(B),** and SOD **(C)** in the presence of 0, 10, and 20 mM H_2_O_2_ treatments. Values are represented as mean ± SD (*n* = 3).

In plant tissues, exogenously applied H_2_O_2_ trigger ROS accumulation leading to oxidative damage. The WT showed significantly higher accumulation of H_2_O_2_ content, on exposure to different H_2_O_2_ concentrations as compared to the transgenics. In WT, 10 mM H_2_O_2_ treatment at 3, 12, and 24 h resulted in higher accumulation of H_2_O_2_ e.g., 197.13, 381.9, and 230.6 mmol/gm FW, respectively. Similarly, at 20 mM H_2_O_2_, WT showed higher accumulation of H_2_O_2_ content at all the duration (182.7, 213.8, and 395.4 mmol/gm FW) compared to transgenics (**Figure 9A**). The CAT enzyme activity enhanced at the early time point (3 h) in both WT and transgenics at 10 mM and 20 mM H_2_O_2_ treatments. However, CAT activity in transgenics was significant low at 12 and 24 h as compared to WT plants. Interestingly, it was observed that transgenic lines (L33, L50, and L64) had significantly higher CAT activity (1127.09, 1286.23 and 1073.01 U/gm FW, respectively) at 3 h during 20 mM H_2_O_2_ treatment as compared to WT (548.3 U/gm FW) (**Figure 9B**).

Another important ROS-scavenging enzyme, SOD, activity of WT with various concentrations of H_2_O_2_ was considerably high as compared to the transgenics. At 10 mM H_2_O_2_ treatment, SOD activity was observed maximum at 12 h (0.41 U/mg FW) and declined at 24 h (0.26 U/mg FW), whereas, with 20 mM H_2_O_2_ treatment gradual increase in activity was observed at 3, 12, and 24 h (0.40, 0.59, and 1.03 U/mg FW, respectively) in WT. There was no significant change in SOD activity in transgenics at both 10 and 20 mM H_2_O_2_ treatments (**Figure 9C**).

### Regulation of Stress-Responsive Downstream Genes in Transgenics

**FIGURE 10:**
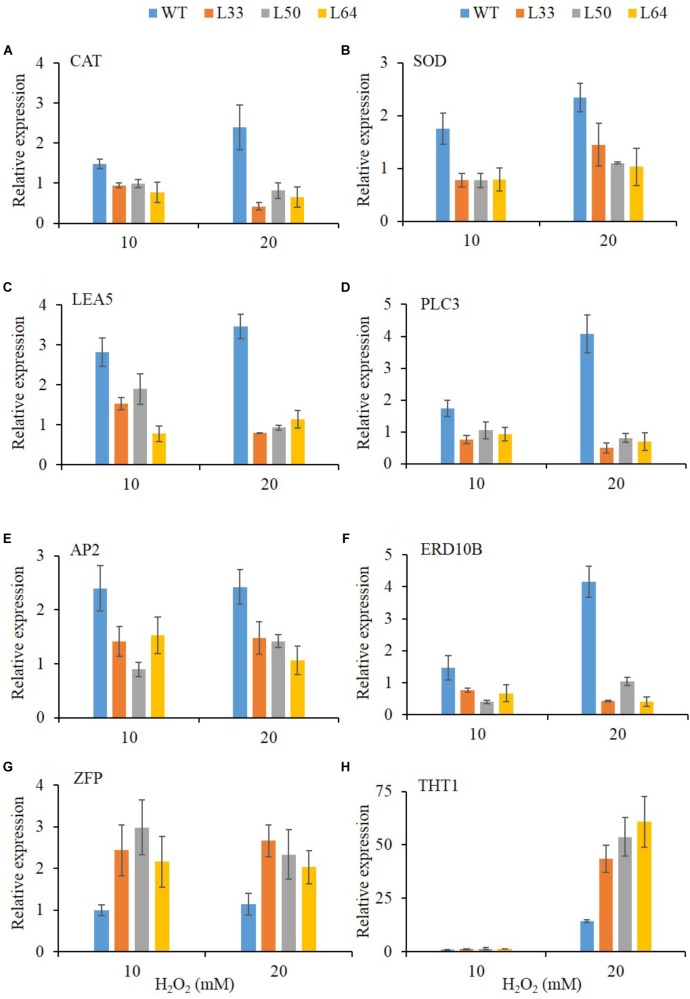
Relative-fold expression of downstream genes in *AlNAC4* transgenics under 0, 10, and 20 mM H_2_O_2_ treatments by Real-Time PCR; **(A)** CAT, **(B)** SOD, **(C)** LEA5, **(D)** PLC3, **(E)** AP2, **(F)** ERD10B, **(G)** ZFP, and **(H)** THT1. Values are represented as mean ± SD (*n* = 3).

To get an insight into the gene regulation of *AlNAC4* transgenics with enhanced H_2_O_2_ tolerance, expression of *CAT* and *SOD* antioxidant genes, stress-related genes like *LEA5*, *PLC3*, *ERD10B*, *THT1* and TFs like *AP2*, *ZFP* were studied. After 24 h, significant accumulation was observed of *CAT* (2.4-fold), *SOD* (2.3-fold), *LEA5* (3.5-fold), *PLC3* (4.1-fold), *AP2* (2.4-fold) and *ERD10B* (4.1-fold) genes at 20 mM H_2_O_2_ in WT plants. All the transgenic lines (L33, L50, and L64) showed no enhanced transcript expression of *CAT*, *SOD*, *LEA5*, *PLC3*, *AP2* and *ERD10B* genes during the same treatments. The higher upregulation of ZFP transcript was observed in transgenics (L33, L50, and L64) during both 10 and 20 mM H_2_O_2_ treatments compared to WT. Whereas, significant upregulation of *THT1* gene (43.4, 53.6, and 60.7-fold) was observed in transgenics (L33, L50, and L64, respectively) at 20 mM H_2_O_2_ treatment compared to WT(14.3-fold) (**Figures 10A–H**).

## Discussion

The NAC superfamily is a largest TF family having multifunctional proteins and play important role in various biological processes and transcriptional regulatory networks. The functions of NACs have been extensively studied in glycophytes but limited studies exist in halophytes. The physiological, biochemical and proteomics studies of *Aeluropus lagopoides* have been carried out to understand its salinity tolerance mechanism (Gulzar and Khan, 2001; Mohsenzadeh et al., 2006; Sanadhya et al., 2015a,b; Sobhanian et al., 2010), however, limited attempts have been made toward isolation and characterization of its stress-responsive genes/TFs. The occurrence of NAC proteins remains restricted to land plants, largely to angiosperms but also includes moss (*Physcomitrella patens*), pteridophytes (*Selaginella moellendorffii*) and conifers (Shen et al., 2009). Here, we identified a NAC gene, *AlNAC4*, from a recretohalophyte *Aeluropus lagopoides*. The AlNAC4 contains a highly conserved NAC domain, which participates in DNA binding, while the C-terminal transcription activation region is highly variable and is considered to be involved in conferring specific response to different environmental stimuli. The NAC domain of AlNAC4 comprises of five sub-domains, [A–E; **Figure 1A**] similar to NAM (*petunia* no apical meristem), ATAF1/2 (*Arabidopsis thaliana* transcription activation factor) and CUC2 (*Arabidopsis* cup-shaped cotyledon) proteins (Souer et al., 1996; Aida et al., 1997). The subdomain A leads to formation of a functional dimer, divergent subdomains B and E provide functional diversity of NAC genes, whereas, the subdomains C and D with a highly conserved positively charged region are responsible for DNA binding (Ernst et al., 2004). The AlNAC4 showed the presence of phosphorylation, glycosylation and other post-translational modification sites. The presence of phosphorylation sites in AlNAC4 suggests the role of phosphorylation in regulating its activity. Phosphorylation is essential for nuclear localization of OsNAC4 (Kaneda et al., 2009), similarly, ATAF1 requires phosphorylation for its subcellular localization, DNA binding activity and protein interactions. Phosphorylation of ZmNAC84 by protein kinase regulates the antioxidant defense in maize (Zhu et al., 2016). The AlNAC4 protein belongs to SNAC group III of stress-responsive NAC genes, as it gets clustered with SNAC group evident from the phylogenetic tree.

The *AlNAC4* showed transcript upregulation by dehydration stress and H_2_O_2_ exposure. Twenty SNAC genes are reported in rice, which are regulated by at least one stress (Fang et al., 2008), similarly, the transcript upregulation of *ZmSNAC1*, *TaNAC4*, *TaNAC69*-1 and *TtNAMB*-2 genes is reported by dehydration and salinity (Baloglu et al., 2012; Lu et al., 2012; Tang et al., 2012).

The AlNAC4 protein show binding to the NACRS of the *erd*1 promoter. Its promoter contains two discrete *cis*-acting elements viz. *myc*-like CATGTG and a 14 bp rps1 site involved in dehydration stress (Simpson et al., 2003). The cDNA of *ANAC015*, *ANAC055*, *ANAC072* were isolated using bait from the *erd*1 promoter (Tran et al., 2004). The NACBS is also reported from several *PR* gene promoters (Seo et al., 2010). The flanking sequence of core binding site in the promoter of the downstream gene also participates in defining the specificity of binding of NAC TF. The ATAF1 protein binds to the promoters of *ORE1* and *GLK1* genes encoding chloroplast maintenance and senescence-promoting TFs, respectively, and promote activation of *ORE1* and repression of *GLK1* transcription (Garapati et al., 2015). NAC TF bind to a wide array of downstream genes to regulate varied biological functions.

The overexpression of *AlNAC4* showed improved resistance toward oxidative stress in tobacco, however, no resistance was observed with salinity or dehydration stress. The low H_2_O_2_, (10 mM)_,_ concentration generated ROS, resulting in the participation of ROS as signaling molecules, and thereby, not much difference was observed in WT and transgenics. However, higher H_2_O_2_ concentration resulted in oxidative stress, whereby, the WT accumulated higher H_2_O_2_ as compared to transgenics during seedling (11-day-old) and 1-month-old stage. Similarly, no difference in germination rate and growth was observed among WT and transgenics at low H_2_O_2_, whereas, at higher concentration, the germination and growth of WT was inhibited significantly. The H_2_O_2_ treatment during seedling stage (11-day-old) was given in Milli-Q water, although it might be variable to stress imposed during natural environment. It is reported that slight increase of ROS serves as signal molecules to activate reversible signal transduction to allow adaptation, whereas, higher levels of ROS cause oxidative stress (Schieber and Chandel, 2014). The NAC TF ZmNTLs is reported to regulate ROS and provide stress tolerance (Wang et al., 2016). The *SNAC3* and *OsNAC2* provide abiotic stress tolerance by modulating the ROS levels, the OsNAC6 activates expression of peroxidases, involved in defense response toward oxidative stress (Nakashima et al., 2007; Fang et al., 2015; Shen et al., 2017). The *ONAC022* confers tolerance against salinity and drought in rice by ABA signaling (Hong et al., 2016). Similarly, wheat *TaNAC29* (Xu et al., 2015) and *Thellungiella halophila* STRESS RELATED NAC1 (*TsNAC1*, Liu et al., 2018) imparts tolerance to salinity in *Arabidopsis*. The LcNAC1 protein upregulates the expression of *LcAOX1a* gene, associated with ROS regulation and energy metabolism (Jiang et al., 2017). The H_2_O_2_-inducible JUNGBRUNNEN1 TF (JUB1, NAC TF), lowers the intracellular H_2_O_2_ levels by regulating different ROS responsive genes in *Arabidopsis* (Wu et al., 2012).

The elimination of excess ROS from plants protect cell and sub cellular systems from cytotoxic effect by involving ROS scavenging antioxidant systems (Mittler et al., 2004). The *AlNAC4* transgenics showed low CAT and SOD activity at both low and high concentrations of H_2_O_2_, as compared to WT. In transgenics, the CAT activity was maximum at 3 h of treatment and consequently reduced with time, however, the WT plants did not show reduction with time. Hsieh et al. (2002) reported that increased catalase activity was associated with decreased H_2_O_2_ content in transgenic tomato overexpressing CBF1 TF. The H_2_O_2_ potential toward promoting seed germination of cereal plants such as barley, wheat and rice (Naredo et al., 1998) and *Zinnia elegans* (Ogawa and Iwabuchi, 2001) by removing the antioxidants inhibitors is reported. Interestingly, the maize ZmNAC84 is phosphorylated by H_2_O_2_ responsive CCaMK (calcium/calmodulin-dependent protein kinase) and this interaction promotes H_2_O_2_ amplification and further, regulating the antioxidant defense activity (Zhu et al., 2016).

The higher tolerance of transgenics is possible due to the regulation of the expression of the different downstream genes. The *AlNAC4* transgenics showed lower expression of anti-oxidative genes (*CAT*, *SOD*), signal transduction proteins, dehydrins, and *AP2* TF. The lower expression of *CAT*, *SOD*, *PLC3*, *LEA5* and *ERD10B* in transgenic lines could be possible as these plants perceive less oxidative stress compared to WT plants by limiting the ROS generation. However, the *AlNAC4* transgenics showed upregulation of *ZFP* and *THT1* genes in presence of H_2_O_2._ The *ZFP182* transient gene expression analysis in rice protoplast suggested its role in ABA-induced anti-oxidative defense (Zhang et al., 2012), also overexpression of *AtRZFP* enhanced tolerance to salt and osmotic stress with reduced ROS accumulation (Baek et al., 2015). THT1 (Hydroxycinnamoyl-CoA:tyramine N-hydroxycinnamoyl transferase) is an important enzyme involved in synthesis of HCAA (hydroxycinnamic acid amides). The HCAA show antioxidant property and is involved in plant development and defense response (Campos et al., 2014).

## Conclusion

We conclude that *AlNAC4* is an important stress-responsive gene, as evident by higher transcript accumulation under dehydration and oxidative stress conditions, and thus fall in the stress-related NAC III (SNACIII) group of largest NAC TF family. The binding of AlNAC4 recombinant protein with the EARLY RESPONSIVE TO DEHYDRATION STRESS 1 (*erd*1) promoter ascertain its potential in regulating downstream genes for modulating stress tolerance. *AlNAC4* imparts enhanced oxidative stress tolerance via triggering the antioxidant pathways. The positive effect of *AlNAC4* transgenics in promoting resistance to oxidative stresses implies its application in developing stress-tolerant crop plants.

## Author Contributions

JK and PA performed the experiments. JK, PA, and PKA were involved in the analysis of data and manuscript writing. PKA and PA coordinated and designed the experiments. All the authors approved the final manuscript.

## Conflict of Interest Statement

The authors declare that the research was conducted in the absence of any commercial or financial relationships that could be construed as a potential conflict of interest.

## Supplementary Material

FIGURE S1 The sequence represents 936 bp cDNA of AlNAC4 and its amino acid sequence. The NAC domain is 21–174 amino acids. The sub-domains A-E are marked by colored arrows [sub-domain A-red, B-blue, C-green, D-pink, E-black]. The residues targeted for PKC and CKII phosphorylation are indicated with solid circled and dashed circled, respectively. The amidation sites (A) are indicated in boxes. The *N*-myristoylation sites (M) are underlined with solid lines and the *N*-glycosylation site (G) is underlined with a dotted line. The cAMP/cGMP dependent phosphorylation site (cAMP PS) is underlined by dashed bold line.

FIGURE S2 Sequence logos of different NAC genes. The overall height of each stack indicates the conservation of the sequence at a particular position. The height of letters within each stack represents the relative frequency of the corresponding amino acid.

FIGURE S3 **(A)** SDS-PAGE analysis of induced and uninduced AlNAC4 recombinant protein in *E*. *coli* BL21 (DE3) star cells. M marker, Lane 1, 3, 5, 7 induced protein with 2 h of vector alone, 2, 4, and 6 h of AlNAC4 culture. Lane 2, 4, 6, 8 uninduced protein with 2 h of vector alone, 2, 4, and 6 h of AlNAC4 culture. **(B)** Lanes PP represent purified recombinant protein and **(C)** Western blot (WB) of purified AlNAC4.

FIGURE S4 The WT and T_0_ transgenic lines with **(A)** vegetative and **(B)** floral stages.

TABLE S1 Primers sequence list.

TABLE S2 Downstream genes and primer sequences used for expression analysis by Real-time PCR in transgenic tobacco plants.
