# Supplementary material for: AlNAC4 Transcription Factor From Halophyte Aeluropus lagopoides Mitigates Oxidative Stress by Maintaining ROS Homeostasis in Transgenic Tobacco
Source: Front Plant Sci. 2018 Oct 29;9:1522. doi: 10.3389/fpls.2018.01522 (PMC6215862; doi:10.3389/fpls.2018.01522)
Supplement: FIGURE S1 — The sequence represents 936 bp cDNA of AlNAC4 and its amino acid sequence. The NAC domain is 21–174 amino acids. The sub-domains A-E are marked by colored arrows [sub-domain A-red, B-blue, C-green, D-pink, E-black]. The residues targeted for PKC and CKII phosphorylation are indicated with solid circled and dashed circled, respectively. The amidation sites (A) are indicated in boxes. The N-myristoylation sites (M) are underlined with solid lines and the N-glycosylation site (G) is underlined with a dotted line. The cAMP/cGMP dependent phosphorylation site (cAMP PS) is underlined by dashed bold line. [file Presentation_1.PPTX]

## Slide 1
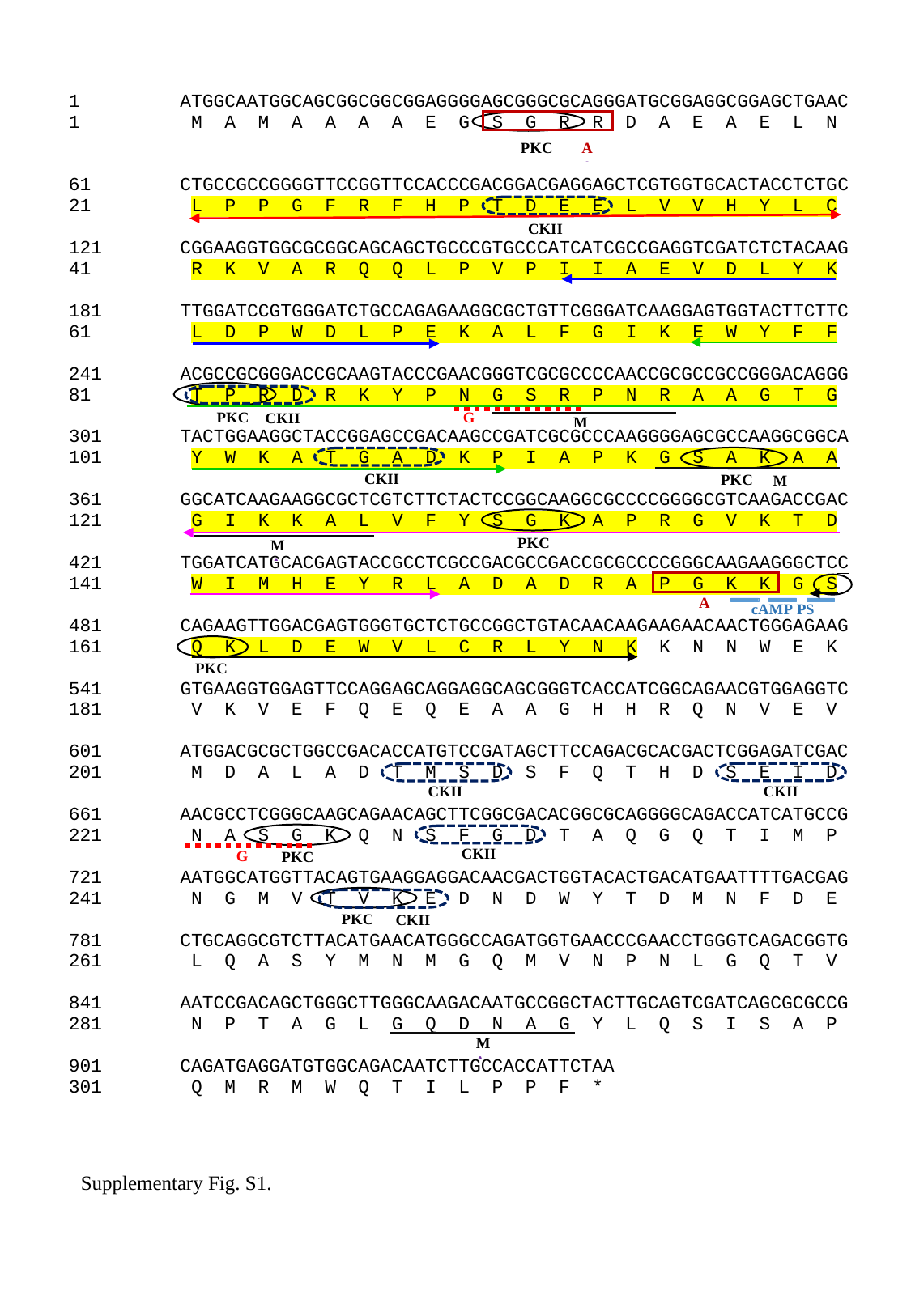

Supplementary Fig. S1.

## Slide 2
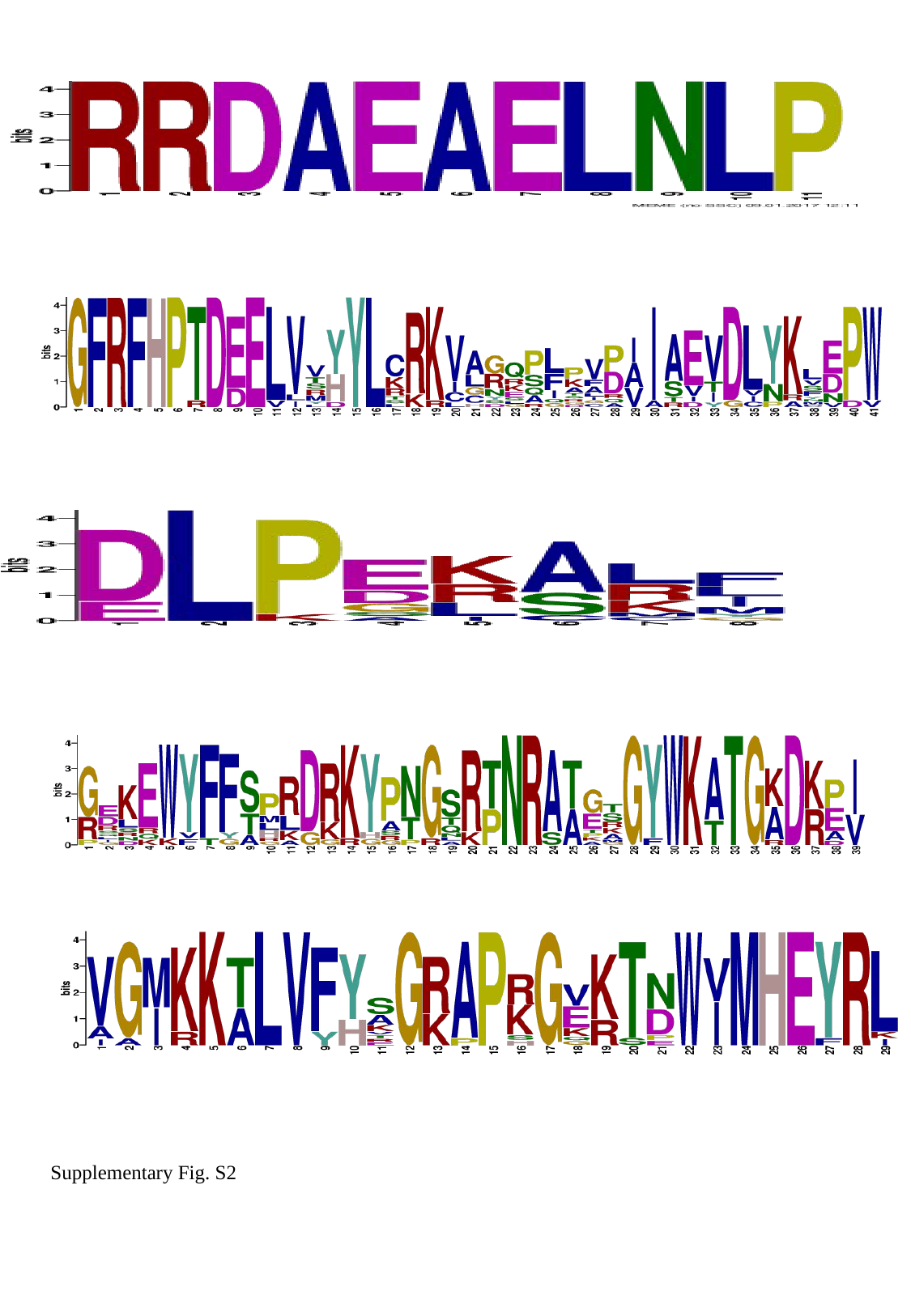

Supplementary Fig. S2

## Slide 3
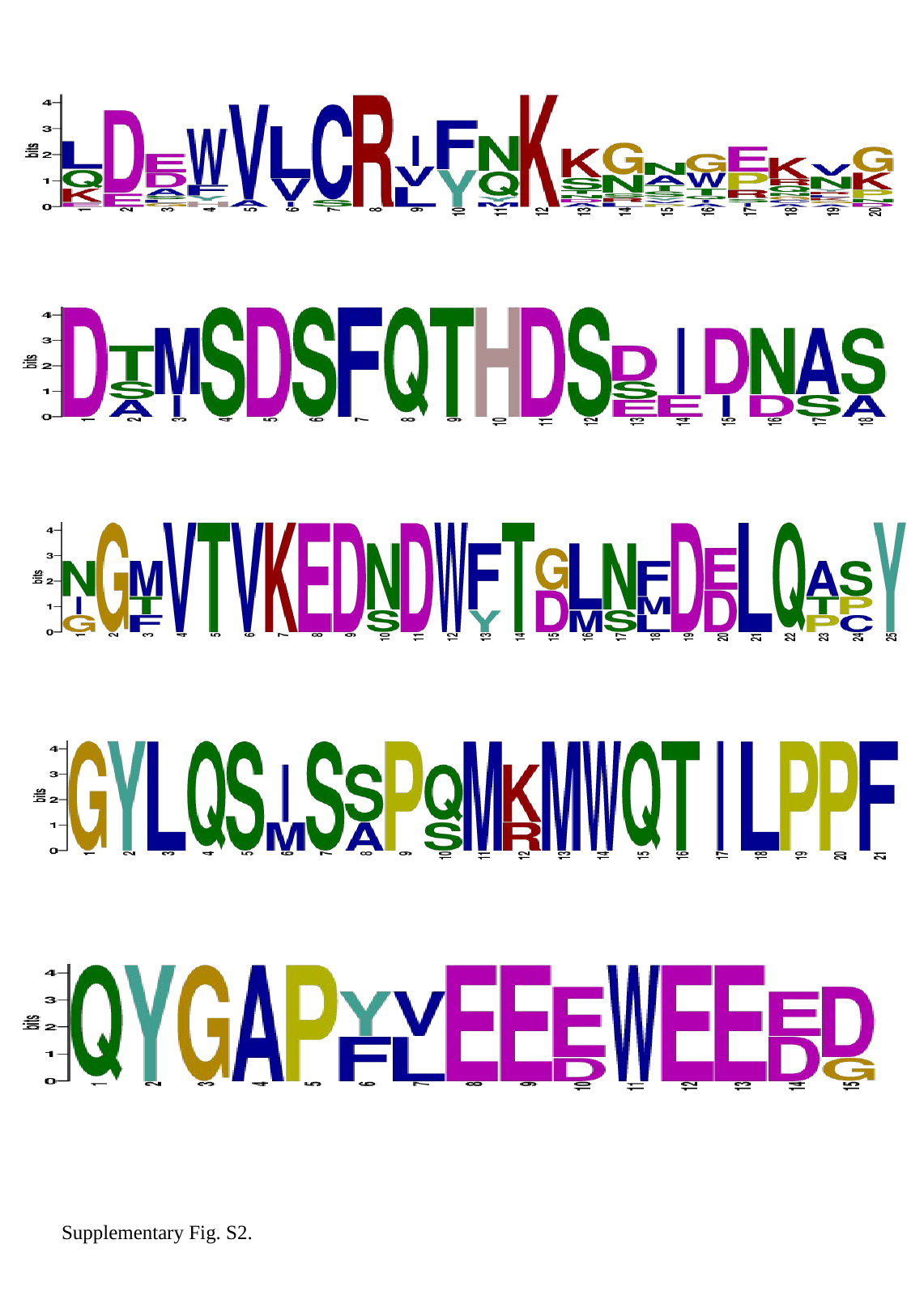

Supplementary Fig. S2.

## Slide 4
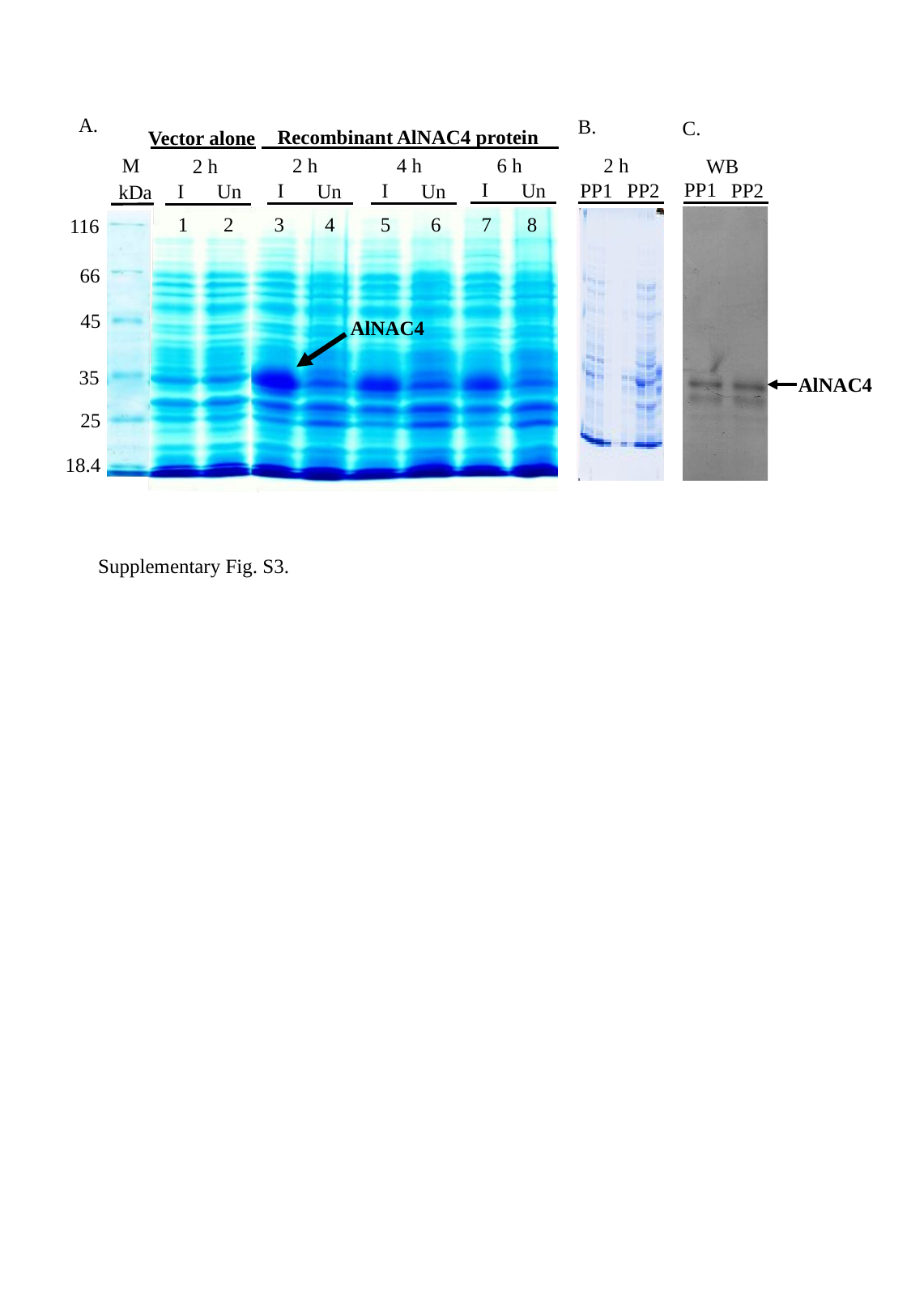

A.
B.
Recombinant AlNAC4 protein
Vector alone
6 h
I
Un
M
kDa
2 h
PP1
PP2
2 h
I
Un
4 h
I
Un
2 h
I
Un
WB
PP1
PP2
1 2 3 4 5 6 7 8
116
66
45
35
25
18.4
AlNAC4
AlNAC4
C.
Supplementary Fig. S3.

## Slide 5
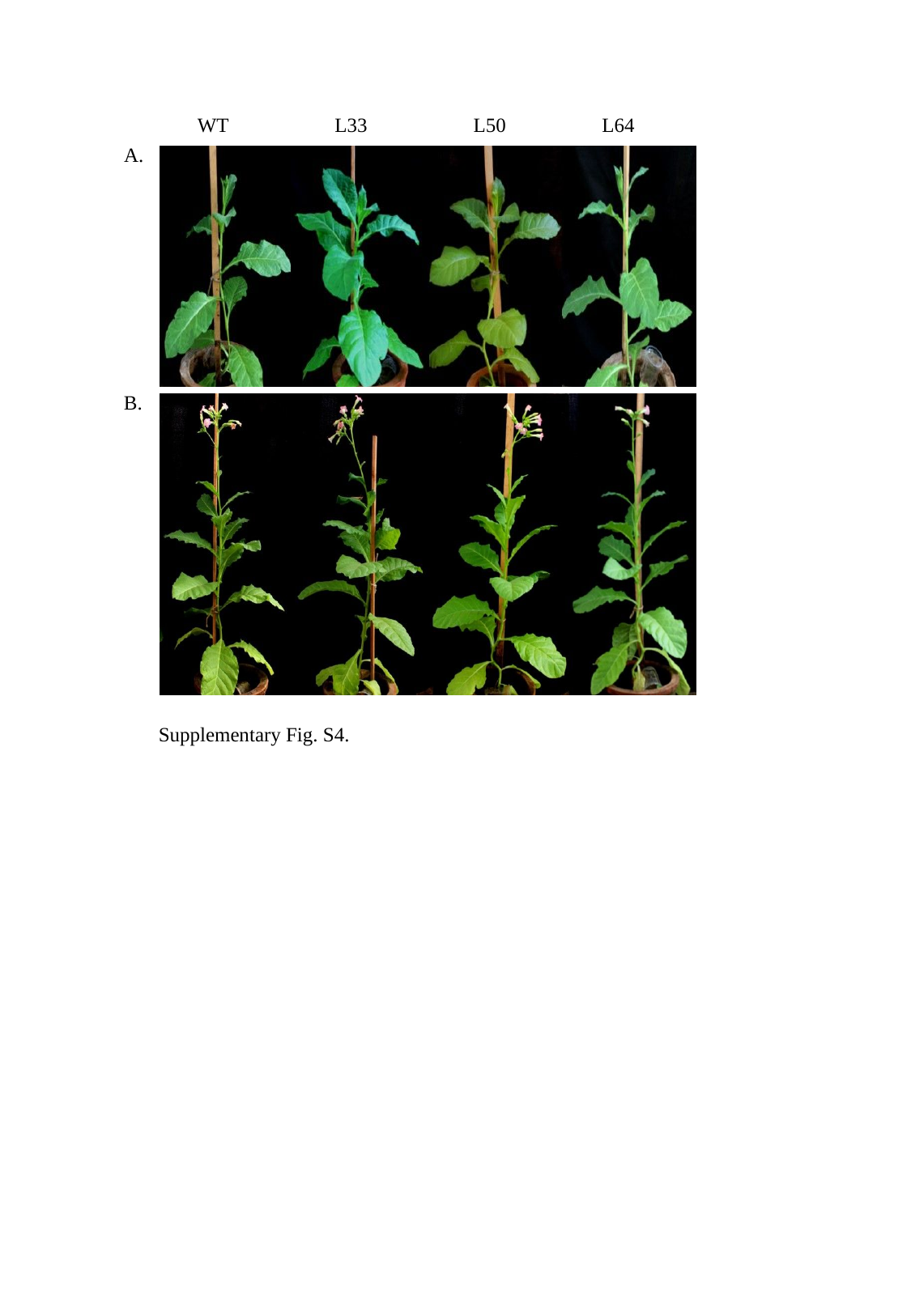

WT L33 L50 L64
A.
B.
Supplementary Fig. S4.
