# Supplementary material for: AlNAC4 Transcription Factor From Halophyte Aeluropus lagopoides Mitigates Oxidative Stress by Maintaining ROS Homeostasis in Transgenic Tobacco
Source: Front Plant Sci. 2018 Oct 29;9:1522. doi: 10.3389/fpls.2018.01522 (PMC6215862; doi:10.3389/fpls.2018.01522)
Supplement: TABLE S1 — Primers sequence list. [file Table_1.DOCX]

Table S1: Primers sequence list

| **Primer** | **Sequence (5’- 3’)** | **Purpose** |
| --- | --- | --- |
| NACFA1 | 5’ CGGAGCTSAACTGCCNCCS 3’ | Degenerate PCR |
| NAC R1 | 5' GYGAKVCKRTACTCRTGCATGATCCA 3' | Degenerate PCR |
| NACFA2 | 5’ CACCCNACNGACGACGAGCT 3’ | Degenerate PCR |
| NAC R2 | 5' GTARAASACBAGSGCCTTCTT SA 3' | Degenerate PCR |
| NACJ5 F1 | 5’ CTGCCCGTGCCCATCATCGC 3’ | 3′ RACE |
| NACJ5 F2 | 5’ GGCGCTGTTCGGGATCAAGGAG 3’ | 3′ RACE |
| NACJ5 F3 | 5’ CACGCCGCGGGACCGCAAGTACC 3’ | 3′ RACE |
| PAoligo-dT | 5’CAGACGAGAGTGTGGAGGACTGCTGCTGGTGTAGC(T)_17_ 3’ | 3′ RACE |
| PAR1 | 5’ CAGACGAGAGTGTGGAGG 3’ | 3′ RACE |
| PAR2 | 5’ GACTGCTGCTGGTGTAGC 3’ | 3′ RACE |
| NACJ5 R1 | 5’ GGTACTTGCGGTCCCGCGGCGTG 3’ | 5′ RACE |
| NACJ5 R2 | 5’ CTCCTTGATCCCGAACAGCGCC 3’ | 5′ RACE |
| NACJ5 R3 | 5’ GCGATGATGGGCACGGGCAG 3’ | 5′ RACE |
| AlNAC4 F | 5’ GCGAGCGAGCCATCCACCCACTC 3’ | Full length PCR |
| AlNAC4 R | 5’ TACATCACTGTCCTCTTCATCTCG 3’ | Full length PCR |
| AlNAC4EcoRI F | 5’ CCGGAATTCATGGCAATGGCAGCGGCGGCGGA 3’ | Cloning |
| AlNAC4XhoI R | 5’ CCGCTCGAGTTAGAATGGTGGCAAGATTGT 3’ | Cloning |
| AlNAC4SalI R | 5’ ACGCGTCGACTTAGAATGGTGGCAAGATTG 3’ | Cloning |
| AlNAC4KpnI R | 5’ CGGGGTACCTTAGAATGGTGGCAAGATTGT 3’ | Cloning |
| AlActin F | 5’ TACGAAGTTTACGCTTCCT 3’ | RT-PCR reference gene |
| AlActin R | 5’ TCTCCAACTCCTCCTCGTAAT 3’ | RT-PCR reference gene |
| AlNAC4RT F | 5’ GATAGCTTCCAGACGCACGAC 3’ | RT-PCR and Transgenic confirmation |
| AlNAC4RT R | 5’ CAAGTAGCCGGCATTGTCTTG 3’ | RT-PCR and Transgenic confirmation |
| NACBST F | 5’ GGCCGCACACGCATGTGACACGCATGTGACACGCATGT  GACACGCATGTGA 3’ | EMSA |
| NACBST R | 5’CTAGTCACATGCGTGTCACATGCGTGTCACATGCGTGTC  ACATGCG TGTGC 3’ | EMSA |
| NACBS F | 5’AGCTTGTGCAACTTGAGCTCTTCTTCTGTAACACGCATGT  GTTGCGTTTGGTTTTTAATTTATTTGCCA 3’ | EMSA |
| NACBS R | 5’ TGGCAAATAAATTAAAAACCAAACGCAACACATGCGTG  TTACAGAAGAAGAGCTCAAGTTGCACAAGCT 3’ | EMSA |
| pBSK+ MCS F | 5’GGGGATCCACTAGTTCTAGAGCGGCCGCCA 3’ | EMSA |
| pBSK+ MCS R | 5’ TGGCGGCCGCTCTAGAACTAGTGGATCCCC 3’ | EMSA |
| gus A F | 5’GAT CGC GAA AAC TGT GGA AT 3’ | Transgenic confirmation |
| gus A R | 5’TGA GCG TCG CAG AAC ATT AC 3’ | Transgenic confirmation |
| hptII F | 5’ TTCTTTGCCCTCGGACGAGTG 3’ | Transgenic confirmation |
| hptII R | 5’ ACAGCGTCTCCGACCTGATG 3’ | Transgenic confirmation |
