# Supplementary material for: AlNAC4 Transcription Factor From Halophyte Aeluropus lagopoides Mitigates Oxidative Stress by Maintaining ROS Homeostasis in Transgenic Tobacco
Source: Front Plant Sci. 2018 Oct 29;9:1522. doi: 10.3389/fpls.2018.01522 (PMC6215862; doi:10.3389/fpls.2018.01522)
Supplement: TABLE S2 — Downstream genes and primer sequences used for expression analysis by Real-time PCR in transgenic tobacco plants. [file Table_2.DOCX]

Table S2: Downstream genes and primer sequences used for expression analysis by Real-time PCR in transgenic tobacco plants

| **S.**  **No.** | **Gene**  **Name** | **Accession no.** | **Primer sequence** | **Description** |
| --- | --- | --- | --- | --- |
| 1. | *NtActin* | XM_016577261.1 | F 5’ GATTTGCTGGTGATGATGCTCC 3’  R 5’ GTCTCAAACATGATCTGTGTCATC 3’ | Actin gene (internal control) |
| 2. | *NtCAT* | HF564632 | F 5’ AGGTACCGCTCATTCACACC 3’  R 5’ AAGCAAGCTTTTGACCCAGA 3’ | Antioxidative enzyme |
| 3. | *NtSOD* | AF443178 | F5’ TGCAGCTCCACCACCAGAAGCATCATCAGAC 3’  R 5’ GGCTCACCACCACCCTCGCGGACA 3’ | Antioxidative enzyme |
| 4. | *NtLEA5* | EFD43044 | F 5’ CCAAACTCATCTCTGCTTTCG 3’  R 5’ AGCATGACTCTCTGGCCTGT 3’ | Signal transduction |
| 5. | *NtPLC3* | EFD43044 | F 5’ TTATGGGTGAAGGGTGGTATTATG 3’  R 5’ GGTCGTGTAGTGAAACTGCTC 3’ | Signal transduction |
| 6. | *NtAP2* | AJ299252 | F 5’ AATACAGAGGAATAAGGCAGAGAC 3’  R 5’ CTCAGCAGCGGGCATTTC 3’ | Signal transduction |
| 7. | *NtERD10B* | AB049336 | F 5’ ACGGACGAATACGGCAATC 3’  R 5’ TCTCCTTAATCTTCTCCTTCATCC 3’ | Dehydrin |
| 8. | *NtZFP* | AF053077 | F 5’ TGCCCCACCGACTGAAGAAGAGTATT 3’  R 5’ GAGGCGGCGGTAGCAGTAGTAGT 3’ | Signal transduction |
| 9. | *NtTHT1* | AJ131768 | F 5’ TAAAGCAAACCCTAATCCTCTC 3’  R 5’ ATTCCTAACTTCCTATAACTCTCC 3’ | Stress elicitors |
